# High-Performance Hydrogen Sensor Fabricated by Layer-by-Layer Self-Assembly for Real-Time Monitoring of Hydrogen Production by Water Electrolysis

**DOI:** 10.3390/nano16171091

**Published:** 2026-09-01

**Authors:** Lan Meng, Rende Zhao, Wenjing Pan, Dongzhi Zhang

**Affiliations:** 1 College of New Energy, China University of Petroleum (East China), Qingdao 266580, China; 13389513533@163.com (L.M.);; 2 State Key Laboratory of Chemical Safety, College of Control Science and Engineering, China University of Petroleum (East China), Qingdao 266580, China

**Keywords:** H_2_ gas sensing, ZnO/Co_3_O_4_ nanocomposite, heterojunction, H_2_ production behavior

## Abstract

In this study, ZnO/Co_3_O_4_ composite thin-film room-temperature hydrogen (H_2_) sensors were prepared via the hydrothermal method and self-assembly techniques. The test results show that the composite sensor with a layer assembly ratio of 1:1 has good sensing performance for H_2_. The ZnO/Co_3_O_4_ composite sensor can detect a wide range of H_2_ concentrations from 100 to 50,000 ppm, and the response toward 300 ppm H_2_ is more than six-times that of a single nanomaterial. In addition, it has a faster response recovery speed, better repeatability, selectivity and long-term stability. The improvement in composite sensor performance results from the synergistic effect between ZnO and Co_3_O_4_ materials and the formation of heterostructures. The prepared ZnO/Co_3_O_4_ composite thin-film sensor was successfully applied to detect the H_2_ production concentration and behavior of an electrolytic cell for hydrogen production by water electrolysis.

## 1. Introduction

Excessive global greenhouse gas emissions have triggered multiple crises, such as climate warming and energy depletion, promoting the low-carbon transformation of the energy system [1]. Hydrogen energy, as a zero-carbon secondary energy carrier, has become one of the core paths to achieve the double-carbon goal [2]. Hydrogen (H_2_) is a colorless, tasteless, and odorless light elemental gas at normal temperature and pressure. Its density is only 1/14 of air, and its diffusion rate is much higher than that of conventional industrial gases [3,4]. The only product of H_2_ combustion or fuel cell electrochemical reaction is water. The entire process does not emit carbon oxides, sulfides or other pollutants, and it has the core advantages of cleanness, efficiency, and regeneration [5]. However, the size of H_2_ molecules is extremely small, the explosion limit is wide, and the minimum ignition energy is extremely low. H_2_ leakage and abnormal H_2_ production concentration during the electrolytic cell gas production process pose major safety risks [6]. Electrolytic water hydrogen production devices still lack real-time H_2_ monitoring methods that can operate at room temperature, have a wide concentration range, and are resistant to humidity interference [7,8]. Existing sensors have high power consumption and are large in size, making it difficult to integrate them into the electrolyzer monitoring systems, which restricts the safe and intelligent management and control of green hydrogen equipment [9,10].

Many metal oxide semiconductors (MOSs) have attracted much attention for gas-sensitive sensing of H_2_ due to their simple manufacturing process, low cost, nanoscale and high responsiveness [11,12]. ZnO is a typical n-type wide-bandgap metal oxide semiconductor with a bandgap width of 3.37 eV, which is non-toxic, has cheap raw materials, and has excellent chemical/thermal stability. It is one of the most mainstream base materials for resistive gas sensors [13]. Chen et al. prepared ZnO/Ti_3_C_2_T_x_ MXene, a heterojunction material with a high specific surface area, through self-assembly technology and achieved ultrafast gas sensing of H_2_ at 80 °C [14]. Sharma et al. used a low-cost hydrothermal process to prepare phase-pure vertically aligned ZnO nanorods. They have excellent performance at 60 ppm H_2_ gas and 100 °C and can achieve ppb-level H_2_ concentration detection [15]. However, the selectivity of a single ZnO nanomaterial is poor, and it needs to be used at a certain operating temperature, with large energy loss and complex equipment [16]. Prior research has shown that constructing heterojunctions can enhance the sensing behaviors of MOS-based gas sensors [17,18].

Cobalt oxide (Co_3_O_4_), as a typical p-type semiconductor sensing nanomaterial, shows excellent selectivity and sensing potential in H_2_ detection. Under normal and medium–low-temperature working conditions, there are a large amount of oxygen adsorption sites on the surface of cobalt oxide. For example, Zhang et al. synthesized bimetallic metal-organic framework-derived Co_3_O_4_/In_2_O_3_ nanocomposite sensors through hydrothermal treatment to monitor triethylamine (TEA), and the sensor delivered a remarkable response of 365.3 when exposed to 20 ppm TEA, which is 30.2-times the response of pure In_2_O_3_ [19]. Yin et al. prepared Pd-SnO_2_-Co_3_O_4_ composites through the hydrothermal method and applied them to H_2_ sensing. The response value of the composite sensor to H_2_ is 57.9, approximately 9.6-fold greater than pristine SnO_2_, and the response time to H_2_ is 12 s, much faster than original SnO_2_. The improvement in H_2_ sensing properties originates from the synergistic effect of p-n junction formation. It can be seen that the modification with Co_3_O_4_ could remarkably strengthen the gas sensing characteristics of the composite sensors and reduce their operating temperature [20].

In this work, the ZnO/Co_3_O_4_ composite film sensor was prepared using hydrothermal preparation combined with a self-assembly process, which achieved the sensitive detection of H_2_ in a wide concentration range at room temperature and demonstrated fast response, response speed, high selectivity and long-term stability. The response performance of the ZnO/Co_3_O_4_ composite film sensor is significantly improved compared to that of a single sensor due to the synergistic effect between ZnO and Co_3_O_4_ and the formation of a heterojunction. Finally, the composite film sensor was successfully used to detect the H_2_ production concentration and behavior of the electrolytic cell of the electrolytic water hydrogen production device, providing a new sensing solution for low-cost, low-power hydrogen monitoring during water electrolysis.

## 2. Experimental Section

### 2.1. Materials

Cobalt nitrate hexahydrate (Co(NO_3_)_2_·6H_2_O, >98.5%), trisodium phosphate (Na_3_PO_4_·12H_2_O, >98%), hydrazine hydrate (N_2_H_4_·H_2_O), zinc nitrate hexahydrate (Zn(NO_3_)_2_·6H_2_O), sodium hydroxide (NaOH), ethanol (C_2_H_6_O, ≥99.7%), polyelectrolyte solvent including 1.5 wt% poly(diallyldimethylammonium chloride) (PDDA) and 0.3 wt% poly(styrene sulfonate) (PSS) were from Aladdin Co. Ltd. (Shanghai, China).

### 2.2. Sensor Fabrication and Test Equipment

Zn(NO_3_)_2_·6H_2_O (2.08 g) was introduced into deionized water (140 mL), followed by ultrasonic stirring for 1 h. Separately, NaOH (3.2 g) was dissolved in deionized (DI) water (20 mL) to yield a NaOH mixed solution. We combined the two solutions and stirred for 1 h. Then, after ultrasonic vibration lasting 30 min, the as-obtained solution was poured into an autoclave and kept at 120 °C for a 12 h hydrothermal reaction to obtain ZnO nanomaterials.

For the preparation of Co_3_O_4_, a certain mass of Co(NO_3_)_2_·6H_2_O was dissolved in ethanol; Na_3_PO_4_·12H_2_O and N_2_H_4_·H_2_O were dissolved in deionized (DI) water, stirred for 1 h, then transferred to a stainless-steel autoclave and heated at 180 °C for 12 h. The Co_3_O_4_ and ZnO solutions were washed several times with DI water to remove excess ions and collected for later use. To enhance the electrostatic interaction between Co_3_O_4_ and ZnO, equal volumes of PDDA and PSS were added to the Co_3_O_4_ and ZnO solutions as cationic and anionic polyelectrolytes, respectively.

**Figure 1:**
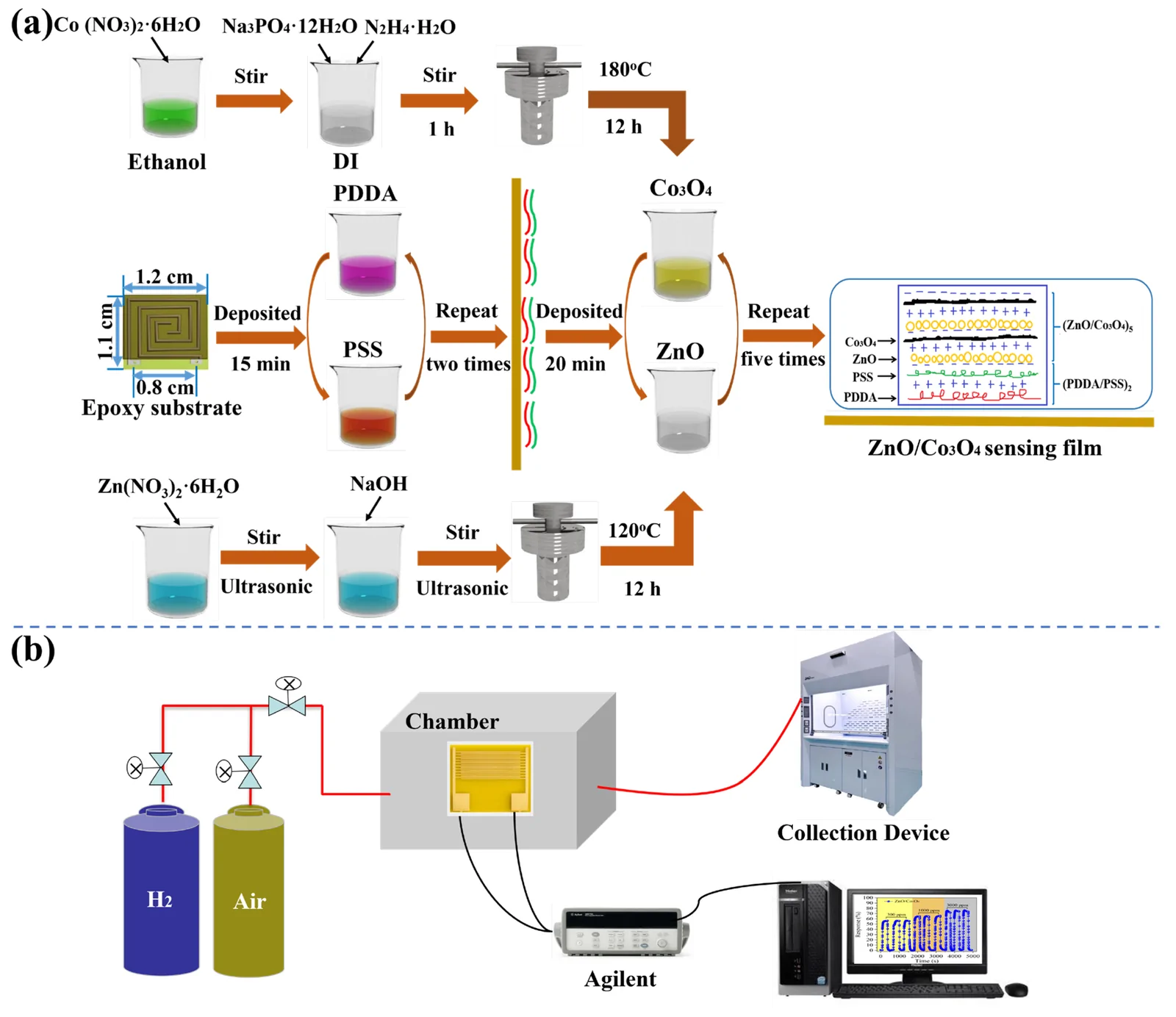
(**a**) Schematic of the preparation process for ZnO/Co_3_O_4_ composite. (**b**) Experimental test device diagram.

Gas sensor based on nanocomposite materials was fabricated via self-assembly approach. A schematic diagram of material preparation and sensor preparation is shown in Figure 1a, which clearly demonstrates the detailed fabrication procedure of this strategy. The interdigital electrode used is shown in Figure 1a, with a length of 1.2 cm, a width of 1.1 cm, and the distance between the two electrodes is 0.8 cm. The interdigital electrode was repeatedly deposited in four different solutions in the figure to obtain the ZnO/Co_3_O_4_ composite sensor. The specific operating steps are as follows. First, immerse the interdigitated electrodes alternately in PDDA and PSS solutions for 15 min each, repeating the process twice to ensure sufficient deposition of the polyelectrolytes onto the electrodes. Then, alternately immerse the electrodes in stirred Co_3_O_4_ and ZnO solutions for 20 min each, repeating this five times to obtain a composite film with a total number of layers of 5, so that Co_3_O_4_ and ZnO can fully combine and firmly deposit onto the interdigitated electrodes under the electrostatic attraction provided by the polyelectrolyte precursor layer. The thin film with different numbers of layers was prepared using layer-by-layer self-assembly technique. The experimental equipment includes Agilent data acquisition instrument, ventilation chamber, H_2_ cylinder, and air cylinder to realize gas concentration configuration and real-time collection of sensor resistance, as shown in Figure 1b. The H_2_ gas detection experiment was conducted at room temperature (25 °C) and 43% relative humidity (RH). Agilent 34970A (Agilent Technologies, Longmont, CO, USA) was used to record the sensor resistance and expose the sensor to different H_2_ concentrations (100–50,000 ppm). The nanocomposite film sensor is suitable for higher-concentration H_2_ sensing. The response value of the sensor follows the formula: R = (R_a_ − R_g_)/R_a_ × 100%, where R_a_ refers to the resistance under ambient air and R_g_ represents the resistance exposed to H_2_ gas.

## 3. Results and Discussion

### 3.1. Material Characterization

**Figure 2:**
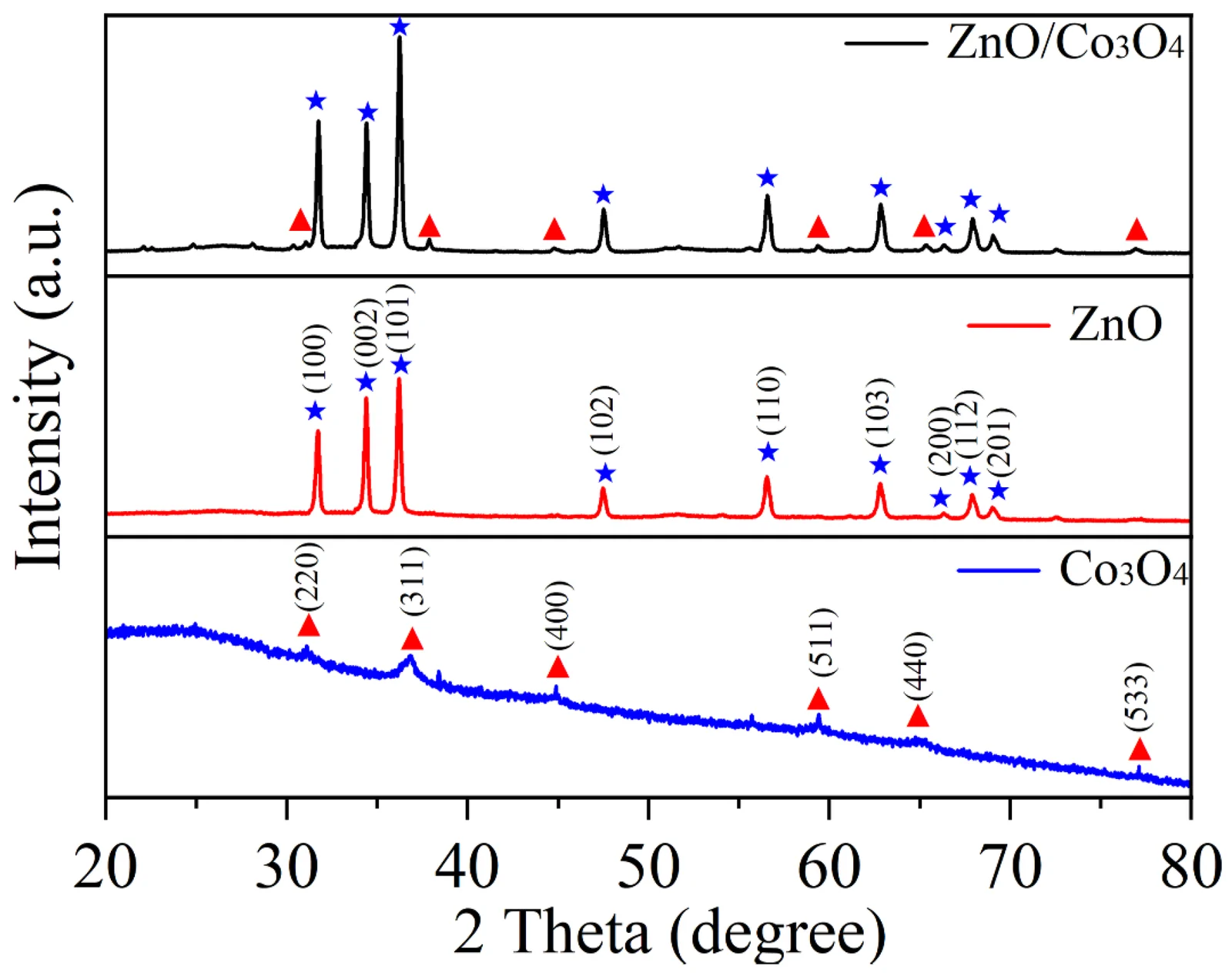
XRD patterns of Co_3_O_4_, ZnO and ZnO/Co_3_O_4_ composite (red triangle indicates the peaks of Co_3_O_4_, and blue star indicates the peaks of ZnO).

XRD patterns were recorded on an X-ray diffractometer (Bruker AXS, Karlsruhe, Germany) equipped with a Cu-Kα radiation source. XRD characterization was performed by compacting and evenly spreading approximately 80 mg of ground ZnO/Co_3_O_4_ composite powder or single nanomaterial powder was collected for characterization. The XRD curves of ZnO, Co_3_O_4_ and ZnO/Co_3_O_4_ composite materials are exhibited in Figure 2, which exhibits the XRD patterns of ZnO, Co_3_O_4_ and ZnO/Co_3_O_4_. The spectrum of ZnO is consistent with the phase spectrum of ZnO nanocrystal planes (JCPDS No. 36-1451) in the standard library, which is at 31.65°, 34.36°, 36.22°, 47.42°, 56.57°, 62.92°, 66.56°, 67.90° and 69.11°, pointing to (1 0 0), (0 0 2), (1 0 1), (1 0 2), (1 1 0), (1 0 3), (2 0 0), (1 1 2) and (2 0 1), respectively [21]. The spectrum of Co_3_O_4_ is consistent with the Co_3_O_4_ in the standard library (JCPDS NO. 43-1003), and the derivatives are at 19.1°, 31.2°, 36.9°, 38.4°, 44.9°, 55.7°, 59.4°, 65.3° and 77.1°. The emission peaks correspond to the (1 1 1), (2 2 0), (3 1 1), (2 2 2), (4 0 0), (4 2 2), (5 1 1), (4 4 0) and (5 3 3) lattice facets of Co_3_O_4_, respectively [22,23]. The XRD patterns of the ZnO/Co_3_O_4_ composite contains the relevant diffraction peaks of ZnO and Co_3_O_4_, confirming the existence of ZnO and Co_3_O_4_ in the nanocomposite.

**Figure 3:**
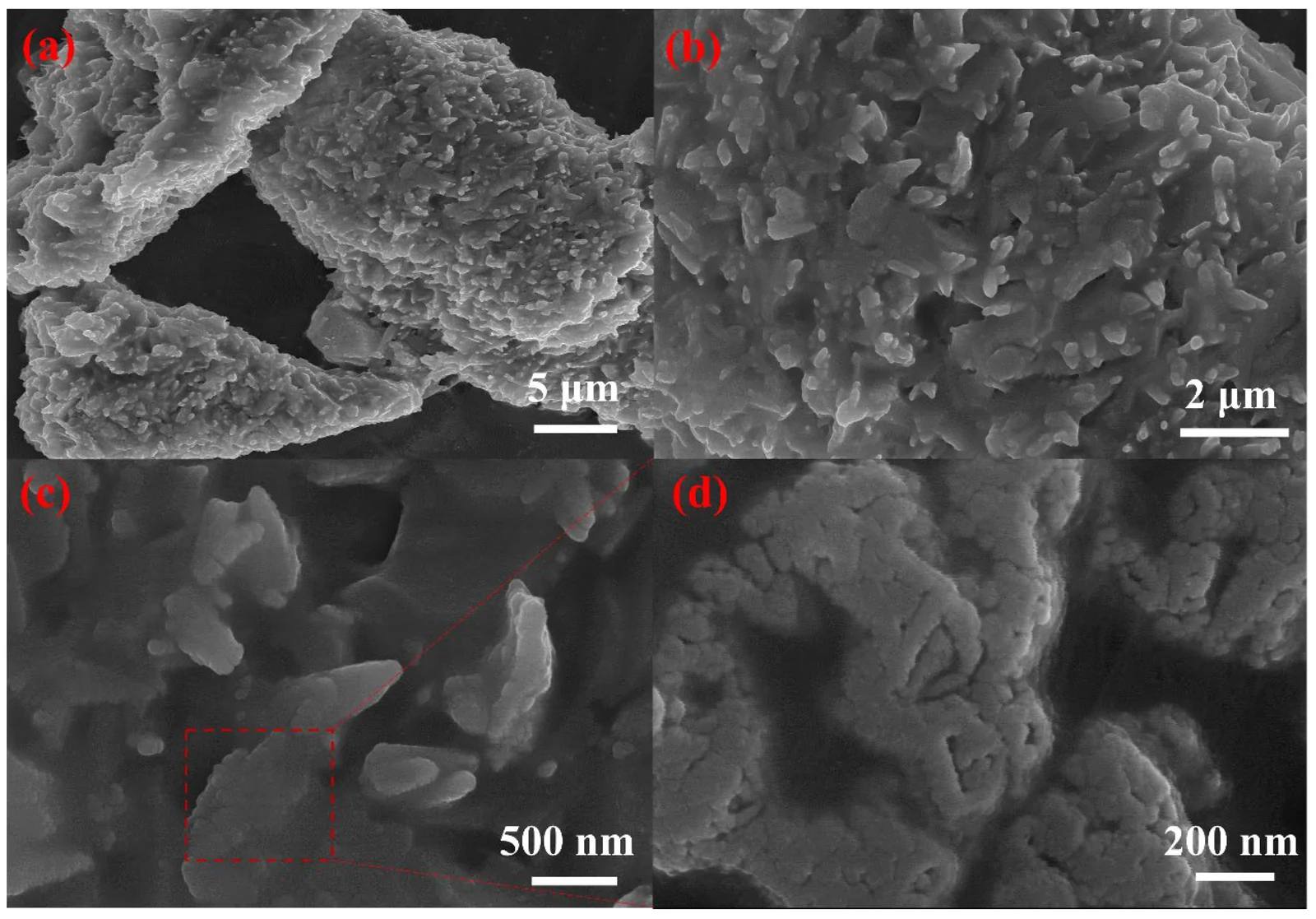
SEM images of (**a**–**c**) ZnO/Co_3_O_4_ nanocomposite, (**d**) ZnO.

SEM characterization was performed using field-emission scanning electron microscopy (FESEM, Hitachi S-4800, Hitachi Ltd., Tokyo, Japan). Figure 3a–c show the morphology of ZnO/Co_3_O_4_ composite material. The SEM image of Co_3_O_4_ can be seen to be similar to an ellipsoid assembled by the agglomeration of nanorods. Figure 3d shows the pristine ZnO is in the shape of cauliflower, composed of nanospheres, tightly wrapped on the spheres composed of Co_3_O_4_ nanorods. From the nanocomposite materials, it is obvious that the two materials are in close and good contact.

**Figure 4:**
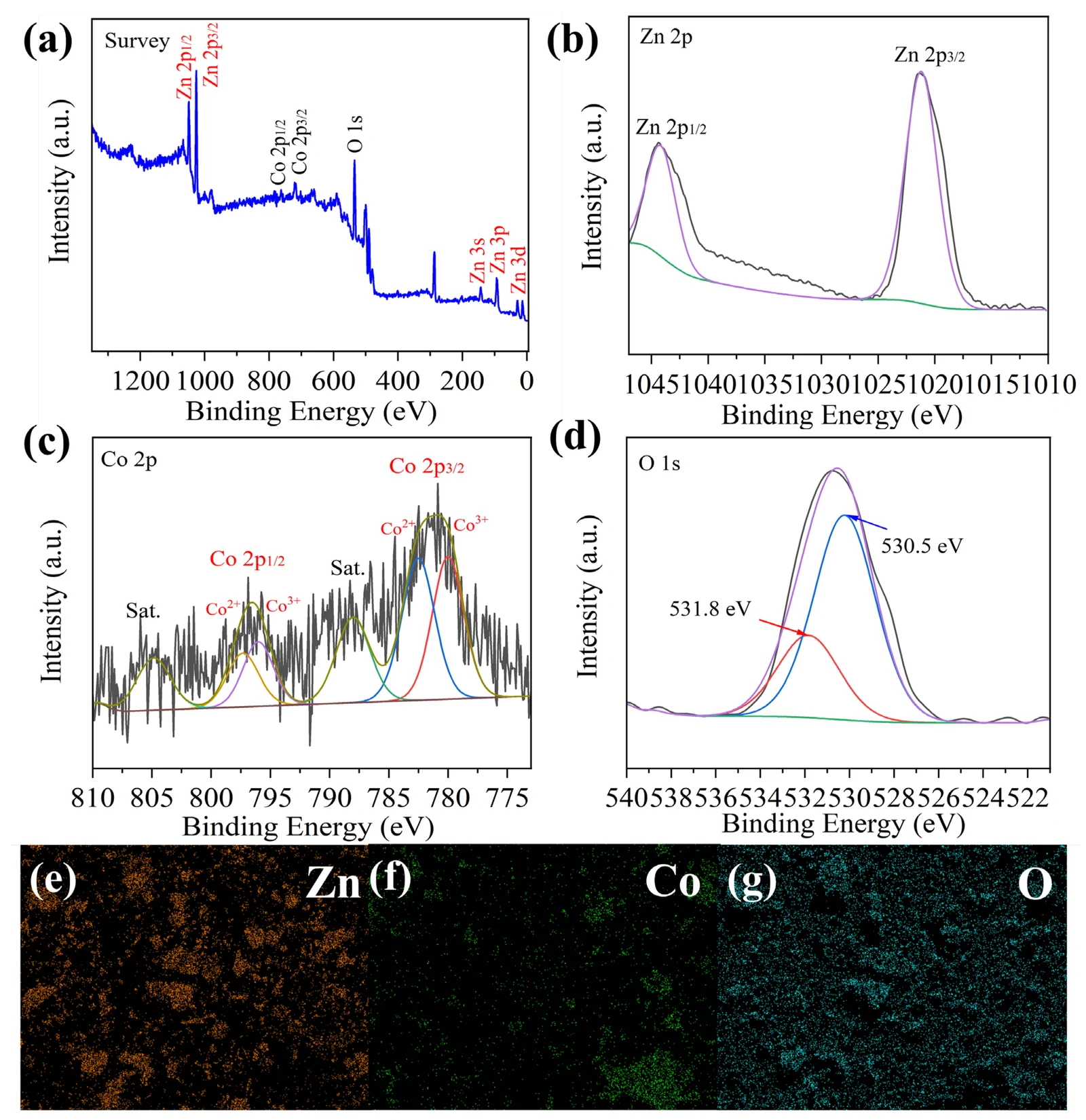
XPS spectra of ZnO/Co_3_O_4_ sample: (**a**) survey spectrum, (**b**) Zn 2p core level spectrum, (**c**) Co 2p core level spectrum, (**d**) O 1s core level spectrum. (**e**–**g**) EDX elemental mapping of Zn, Co, O.

X-ray photoelectron spectroscopy (Thermo Scientific, Waltham, MA, USA) was used to analyze the surface composition and elemental state of ZnO/Co_3_O_4_ nanocomposites. Figure 4a shows the XPS spectrum curve. It can be seen that Zn, Co and O elements exist in the ZnO/Co_3_O_4_ composite material. Figure 4b shows the XPS spectrum of Zn 2p. The two peaks located at 1044.3 eV and 1021.2 eV correspond to Zn 2p_1/2_ and Zn 2p_3/2_, respectively, which indicates that the Zn state in the ZnO/Co_3_O_4_ nanocomposite is normal [14]. In Figure 4c, the XPS spectrum of Co 2p has two obvious peaks at 781.1 eV and 796.5 eV, corresponding to the Co 2p_3/2_ and Co 2p_1/2_ peaks, respectively. Co 2p_3/2_ as the main peak consists of Co^3+^ (780.1 eV) and Co^2+^ (782.5 eV). Co 2p_1/2_ as a shoulder consists of Co^3+^ (795.9 eV) and Co^2+^ (797.2 eV). This proves the successful synthesis and existence of Co_3_O_4_. There are two weak peaks at the binding energies of 787.8 and 804.7 eV, corresponding to the Co^2+^ shake-up satellite peak [24,25]. Figure 4d shows two peaks of O 1s located at 530.5 and 531.8 eV. The former corresponds to the O^2−^ anions in ZnO and the lattice oxygen in the Co_3_O_4_ phase, and the latter corresponds to the O^−^ anions and O^2−^ anions generated by oxygen vacancies [26]. The element distribution and composition of the ZnO/Co_3_O_4_ composite were characterized by energy-dispersive X-ray spectroscopy (EDX). The elements Zn, Co and O are evenly distributed, as shown in Figure 4e–g, which proves the element composition and content.

### 3.2. H_2_ Sensing Properties

**Figure 5:**
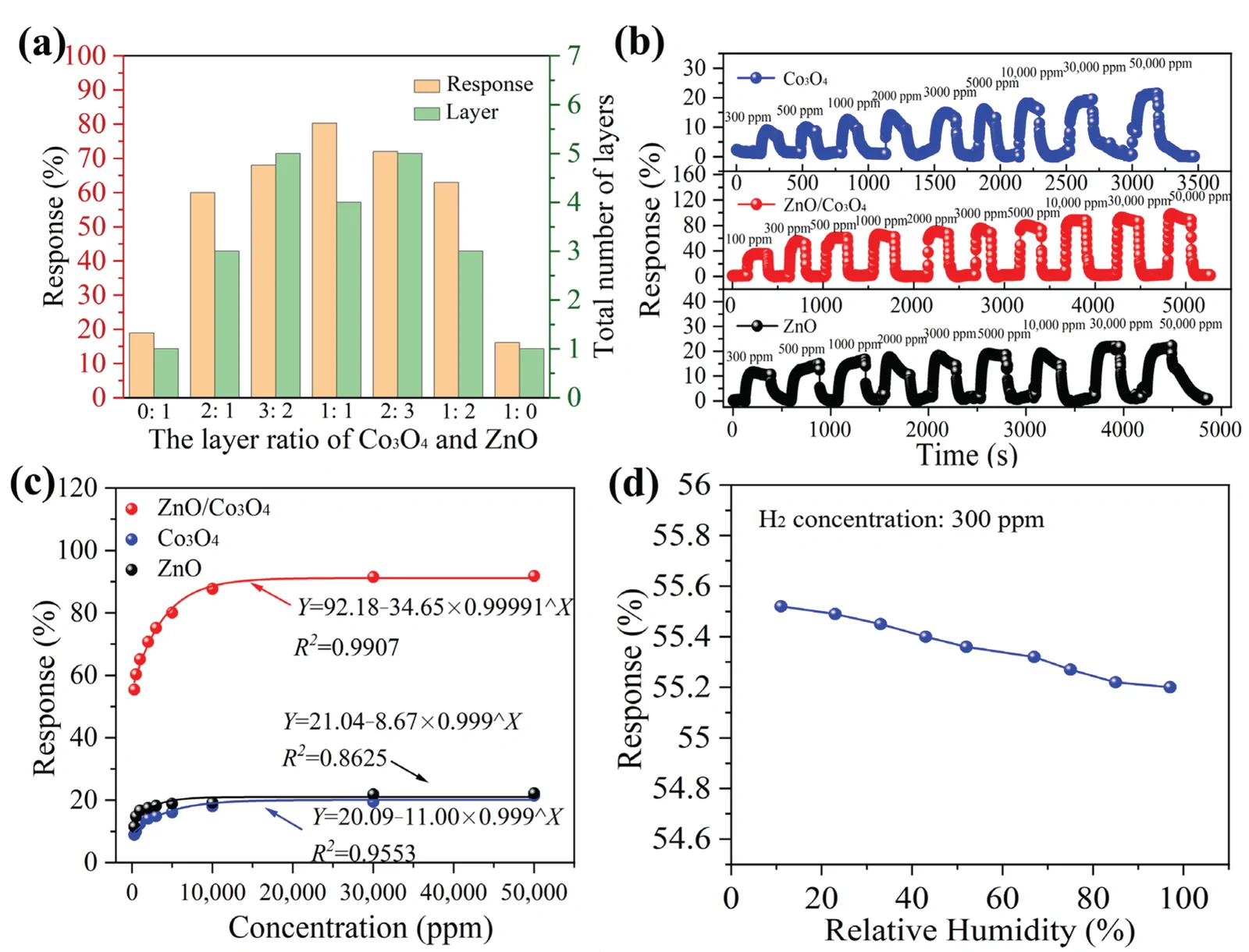
(**a**) The H_2_ sensing performance of ZnO/Co_3_O_4_ composite with different layer ratio of Co_3_O_4_ and ZnO. (**b**) Responses and (**c**) function fitting curves of ZnO/Co_3_O_4_ composite and individual sensors toward various concentrations of H_2_ at 25 °C. (**d**) The effect of relative humidity on the response of ZnO/Co_3_O_4_ composite sensor at 25 °C.

Figure 5a shows the response value of sensors based on different Co_3_O_4_ and ZnO layer ratios to H_2_. Seven sensors featuring distinct component ratios were measured upon exposure to 5000 ppm H_2_ under room-temperature conditions. The responses and total number of layers of the seven sensors are displayed in the form of a histogram. The specific values are displayed on the left and right Y coordinate axes. The total number of assembly layers from left to right is 1, 3, 5, 4, 5, 3 and 1. The results showed that the sample was fabricated at a 1:1 layer ratio, and a total number of four layers delivered the maximum response magnitude. This may be due to the low conductivity of sensors with fewer layers, while gas desorption is more difficult for sensors with more layers [27]. Hence, in subsequent experiments, Co_3_O_4_ and ZnO layers were used to assemble a sensor with a ratio of 1:1 and a total number of layers of 4. The responses of Co_3_O_4_, ZnO and ZnO/Co_3_O_4_ sensors to different H_2_ concentrations are shown in Figure 5b. It is evident that the ZnO/Co_3_O_4_ composite nanofilm has the highest response to H_2_ compared with the other two sensors. The response values of nanocomposite film sensors toward 100–50,000 ppm are 34.9%, 55.4%, 60.3%, 65.1%, 70.7%, 75.2%, 80.1%, 87.7%, 91.5%, 91.8%, respectively. The detection limit of the composite sensor is 100 ppm, which is lower than the detection limit of the single sensor of 300 ppm. It is worth noting that the 1:1 layered ZnO/Co_3_O_4_ nanocomposite sensor achieves a response of 55.4% at 300 ppm H_2_, far exceeding the value of 8.9% recorded for pristine Co_3_O_4_, and the response magnitude is roughly 6.22-fold higher. This is due to the synergistic effect between ZnO and Co_3_O_4_ nanomaterials and the formation of a heterojunction, which can improve the H_2_ sensing performance. It is also worth emphasizing that the H_2_ sensing range for the sensor is from 100 ppm to 50,000 ppm. The sensor has a wide H_2_ sensing detection range and can detect high concentrations of H_2_. Figure 5c displays the fitted curves correlating the response values of the three kinds of sensors with H_2_ concentrations. For ZnO, Co_3_O_4_ and ZnO/Co_3_O_4_ sensors, the fitting functions are Y = 21.04 − 8.67 × 0.999^X^, Y = 20.09 − 11.00 × 0.999^X^ and Y = 92.18 − 34.65 × 0.99991^X^, respectively. The correlation coefficients (R^2^) were 0.8625, 0.9553 and 0.9907, respectively. Figure 5d illustrates the difference in response of the ZnO/Co_3_O_4_ composite sensors toward 300 ppm H_2_ at different relative humidity. It can be seen that when the relative humidity increases from 11% to 97%RH, the sensor response changes slightly within the range of 0.4%, indicating good humidity resistance.

**Figure 6:**
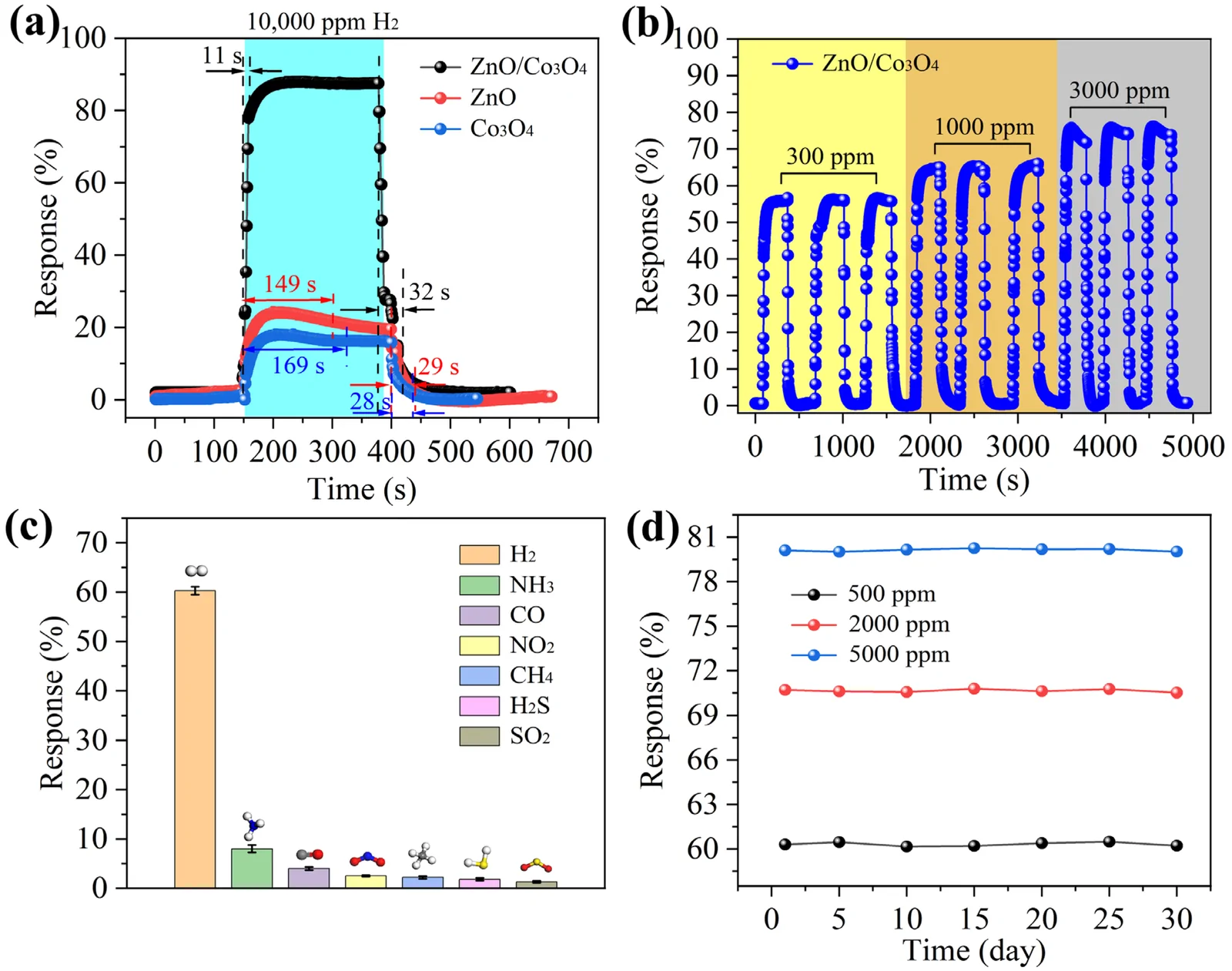
(**a**) Response–recovery curves of ZnO/Co_3_O_4_ composite and individual sensors toward 10,000 ppm H_2_. (**b**) Repeatability of the ZnO/Co_3_O_4_ composite sensor upon exposure to 300, 1000 and 3000 ppm H_2_. (**c**) Selectivity of the ZnO/Co_3_O_4_ composite sensor toward different interfering gases of 500 ppm. (**d**) Long-term stability of the ZnO/Co_3_O_4_ composite sensor upon exposure to 500, 2000 and 5000 ppm H_2_ at 25 °C.

Figure 6a presents the response–recovery behaviors of pure and composite sensors upon exposure to 10,000 ppm H_2_. The response and recovery durations of Co_3_O_4_, ZnO and ZnO/Co_3_O_4_ sensors are 169 s/28 s, 149 s/29 s and 11 s/32 s, respectively. Obviously, the ZnO/Co_3_O_4_ sensor exhibits a faster response than a single-material sensor. Figure 6b shows the repeatability of the ZnO/Co_3_O_4_ composite sensors under 300, 1000 and 3000 ppm H_2_, demonstrating outstanding uniformity over three repeated measurement cycles for each concentration. Figure 6c exhibits the selectivity of the ZnO/Co_3_O_4_ composite sensor to 500 ppm hydrogen (H_2_), ammonia (NH_3_), carbon monoxide (CO), nitrogen dioxide (NO_2_), methane (CH_4_), hydrogen sulfide (H_2_S), and sulfur dioxide (SO_2_) at room temperature. It is evident that the selectivity of the ZnO/Co_3_O_4_ nanofilm sensors toward H_2_ gas is remarkably higher than that of other gases, which can reach more than six-times. The long-term stability measurement results of ZnO/Co_3_O_4_ nanocomposites at 500, 2000 and 5000 ppm H_2_ are shown in Figure 6d. The tests were conducted over a month, with 5 days between each test. It can be observed from the diagram that the ZnO/Co_3_O_4_ sensor possesses outstanding repeatability and favorable long-term stability.

The last row of Table 1 summarizes the H_2_ sensing characteristics of the ZnO/Co_3_O_4_ nanofilm sensor in this study and compares it with H_2_ sensors in recent years, including the most recent year in existing research [28,29,30,31,32,33,34,35,36,37,38,39,40,41]. The results show that the proposed ZnO/Co_3_O_4_ sensor achieves outstanding room-temperature H_2_ response that outperforms the H_2_ sensing devices reported so far in Table 1. At present, most H_2_ sensors work at a certain operating temperature. In this study, the room-temperature gas sensor can greatly lower the power consumption, which can make it more lightweight and convenient.

### 3.3. H_2_ Gas Sensing Mechanism

**Figure 7:**
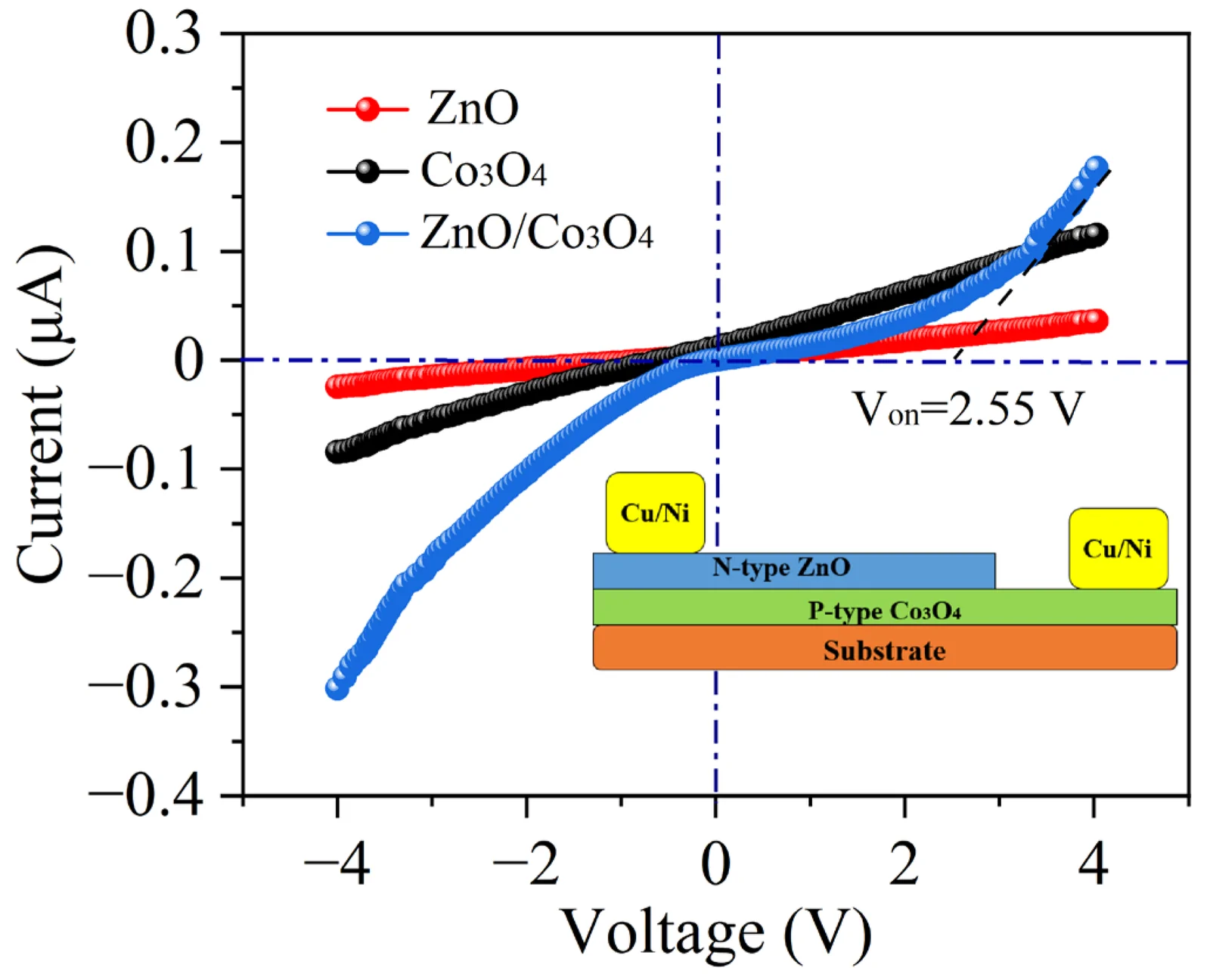
Current–voltage curve of pure ZnO, Co_3_O_4_ and ZnO/Co_3_O_4_ composite-based sensors.

Keithley-2400 (Keithley Instruments, Solon, OH, USA) is used to test the current–voltage (I-V) curves of three types of sensors, using a voltage of −4 V to 4 V for scanning: ZnO, Co_3_O_4_ and ZnO/Co_3_O_4_ composite materials. Pristine ZnO and Co_3_O_4_ sensors exhibit linear current–voltage characteristics, as illustrated in Figure 7. Differently, the ZnO/Co_3_O_4_ heterostructure shows reverse saturation of junction current in the negative-bias region, whereas positive-bias current undergoes near-exponential growth. Such electrical behaviors confirm the prominent nonlinear rectification property of the ZnO/Co_3_O_4_ composite heterojunction [42,43]. The turn-on voltage is approximately 2.55 V. The electrode schematic is shown in the figure inset.

From the test, it can be found that the resistance of the ZnO/Co_3_O_4_ film sensor decreases in H_2_ gas, indicating that the composite film sensor is an n-type semiconductor [44]. The ZnO/Co_3_O_4_ composite film sensor has good sensing performance for H_2_ at room temperature. Due to the good combination of the two nanomaterials, the ZnO/Co_3_O_4_ heterostructure offers plentiful reactive sites for H_2_ adsorption, playing a vital role in optimizing and promoting the H_2_ sensing behavior. When the nanofilm is in the air, the following Reactions (1) and (2) occurs. When the sensor is in H_2_, the following Reaction (3) occurs, while reducing the resistance of the composite film [45,46].

O_2(gas)_ → O_2(ads)_ (1)

O_2(ads)_ + e^−^ → O_2_^−^ _(ads)_ (2)

H_2(ads)_ + O_2_^−^ → H_2_O_(g)_ + e^−^ (3)

**Figure 8:**
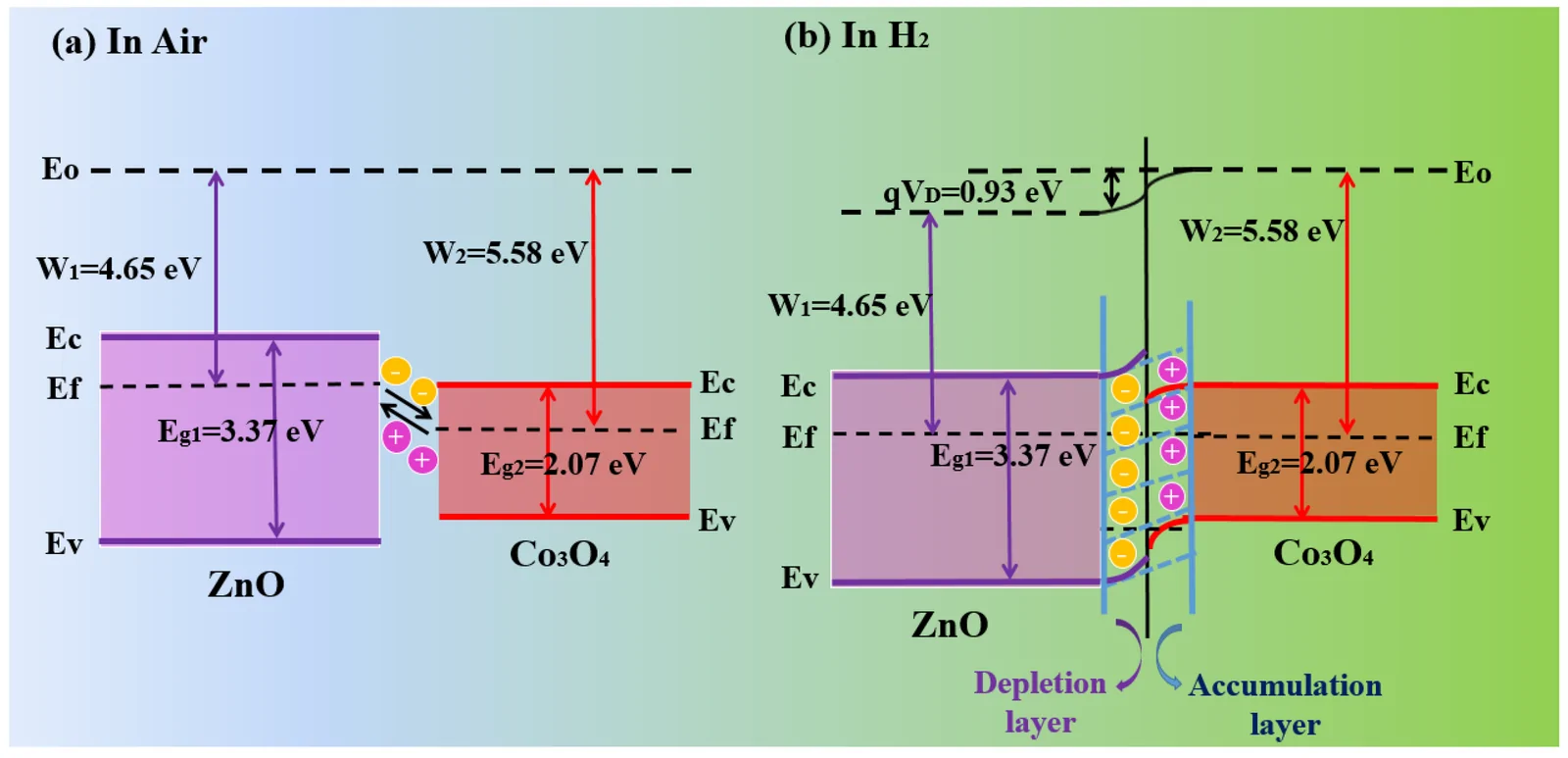
Schematic diagram of the gas sensing mechanism and energy band structure of ZnO/Co_3_O_4_ composite in (**a**) air and (**b**) H_2_ gas.

The H_2_ sensing performance of ZnO/Co_3_O_4_ films is remarkably better than that of pure ZnO and Co_3_O_4_, which can be ascribed to the synergistic effect between ZnO and Co_3_O_4_. The energy band diagrams of n-type ZnO and p-type Co_3_O_4_ are shown in Figure 8a. The energy gaps of ZnO and Co_3_O_4_ are approximately 3.37 and 2.07 eV [47]. ZnO is tightly wrapped on the surface of Co_3_O_4_, which helps to form a heterojunction at the interfacial region. Meanwhile, the work functions of ZnO and Co_3_O_4_ are different, which are 4.65 eV and 5.58 eV, respectively. Because the Co_3_O_4_’s work function is higher than that of ZnO, the Fermi level of Co_3_O_4_ is located in the lower part of ZnO. Therefore, electrons are transferred from the valence band of ZnO to the conduction band of Co_3_O_4_ to balance its Fermi level [48]. Figure 8b presents the energy band mechanism of the ZnO/Co_3_O_4_ heterojunction under H_2_ ambient conditions. Due to the formation of the heterogeneous interface between ZnO and Co_3_O_4_, H_2_ molecules can be readily adsorbed on the composite surface. On the one hand, the formation of p-n heterojunctions between ZnO and Co_3_O_4_ accelerates electron transfer and facilitates the rapid oxidation of H_2_ molecules [49]. On the other hand, the heterojunction structure suppresses electron–hole recombination, enabling more electrons to be efficiently transferred from H_2_ molecules to the surface of sensing nanomaterials [50]. Therefore, the interfacial heterojunction of ZnO and Co_3_O_4_ is responsible for the prominently enhanced H_2_ sensing performance of the ZnO/Co_3_O_4_ composite sensor.

### 3.4. Hydrogen Production Test with Electrolyzing Water

**Figure 9:**
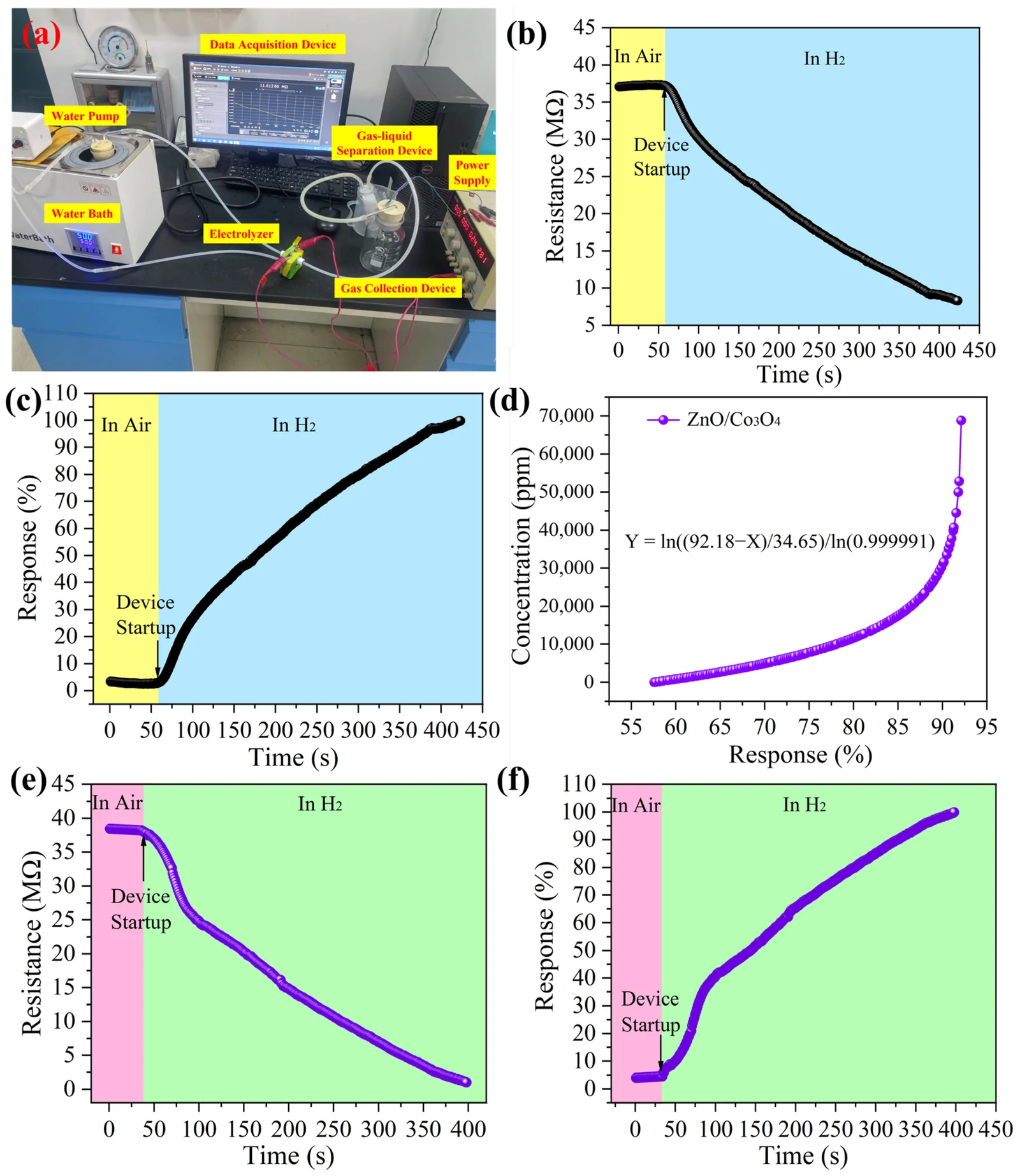
(**a**) H_2_ gas production and H_2_ gas testing experimental device using water electrolysis hydrogen production device. (**b**) Resistance changes of the composite film sensor before and after the start-up of the water electrolysis hydrogen production device. (**c**) Changes in the response value of the composite film sensor before and after the start-up of the water electrolysis hydrogen production device. (**d**) Functional relationship between composite film sensor response and H_2_ gas concentration during hydrogen production. Changes in (**e**) resistance and (**f**) response value of the composite film sensor before and after the start-up of the electrolytic water hydrogen production device in another time period.

The hydrogen production via water electrolysis and using a hydrogen testing device is shown in Figure 9a, which mainly consists of a water bath, a water pump, an electrolyzer, a gas–liquid separation device, a power supply, a gas collection device and a data acquisition device. The electrolytic cell is powered by a power supply, and the positive and negative clips of the data acquisition device clamp both ends of the sensor electrode connection to record the sensor resistance value in real time. The H_2_ generated by electrolyzing water in the electrolyzer passes through the tube, passes through the gas–liquid separation device in the middle, and continuously flows into the gas collection container. The testing process is as follows: the H_2_ gas produced by electrolyzing water in the electrolytic cell is passed into the gas collection device through a tube, and a gas–liquid separation device is added in the middle to avoid the influence of greater humidity caused by the moisture in the H_2_ gas produced by electrolyzing water. Using the power supply to the electrolyzer, the H_2_ production process begins.

The initial resistance value of the composite sensor is about 37 MΩ. After the hydrogen production device by water electrolysis is started, as H_2_ gas continues to be introduced, the resistance of the composite film sensor continues to decrease, as shown in Figure 9b. The response is calculated using (R_air_ − R_gas_)/R_air_ × 100% as shown in Figure 9c. The sensor response increases as H_2_ gas is introduced for a longer time. This is because the concentration of H_2_ gas continues to accumulate and increase as the gas introduction time increases. The functional relationship between the sensor response and H_2_ concentration in this process is shown in Figure 9d. This expression can be used to monitor the changes in H_2_ concentration during the H_2_ production process, thereby monitoring H_2_ production in real time. For example, it can be observed from Figure 9c that the sensor response value achieves 91.8% when the H_2_ gas is passed for about 300 s, and the corresponding H_2_ concentration is 50,000 ppm. The gas concentration calculation formula in ppm is C_ppm_ = V_gas_/V_total_ × 10^6^, and the volume of the gas collection container is 500 mL, so the volume of H_2_ flowing into the gas collection container can be calculated as 25 mL, and the instantaneous H_2_ production rate of the electrolytic cell at this time can be further calculated as about 5 mL/min. In practical electrolysis tests, hydrogen gradually accumulates from low concentration to 50,000 ppm within 300 s, and most of the test process falls in the 100–30,000 ppm accurate quantitative range, with a high R^2^ of 0.9907 for accurate concentration calculation. The stable saturated response platform at 50,000 ppm acts as a definite concentration endpoint reference. The resistance and response curves of the composite film sensor tested in another time period before and after the start-up of the electrolytic water hydrogen production device are shown in Figure 9e,f, demonstrating real-time monitoring of hydrogen production by water electrolyzers was achieved using the prepared composite sensor.

## 4. Conclusions

In this work, hydrothermal synthesis coupled with layer-by-layer self-assembly was adopted to fabricate ZnO/Co_3_O_4_ composite films for high-performance room-temperature H_2_ detection. The p-n heterojunction formed at the ZnO-Co_3_O_4_ interface and the synergistic effect of the two metal oxides jointly contribute to the prominent promotion of comprehensive sensing performance, covering elevated response signal, rapid response/recovery speed, excellent cycling repeatability and long-term stability relative to pure ZnO and Co_3_O_4_. More importantly, the developed composite sensor was practically applied into a water electrolysis hydrogen production platform, realizing real-time measurement of hydrogen concentration and evaluation of electrolytic hydrogen production behavior.

## Figures and Tables

**Table 1:** Comparison of H_2_ gas sensing performance in previous reports with that of this work.

Sensing Material	t_res_/t_rec_	Temperature	Response (%)	Ref.
SnO_2_/Co_3_O_4_	3 s/18 s	325 °C	16.56@100 ppm	[28]
Pd_8_SR_16_-SDBS-rGO	1 s/6 s	RT	4.48@1000 ppm	[29]
TiO_2_ QDs-SnO_2_	2 s/5 s	400 °C	40.6@200 ppm	[30]
Au–Pd/SnO_2_	8 s/12 s	100 °C	220@100 ppm	[31]
Pd/WO_3_	10 s/15 s	150 °C	200@100 ppm	[32]
Pd,Ce/WO_3_ nanorods	9 s/14 s	120 °C	180@100 ppm	[33]
Pd-NiO	20 s/25 s	150 °C	120@100 ppm	[34]
Pd-GaN nanowires	30 s/40 s	250 °C	60@100 ppm	[35]
Pd/NiCo_2_O_4_ nanoneedles	15 s/20 s	150 °C	100@100 ppm	[36]
Pd NP/Si nanoforest	12 s/18 s	150 °C	110@100 ppm	[37]
Au-ZnO	9.26 s/24.3 s	230 °C	37.6@100 ppm	[38]
Co_3_O_4_/SnO_2_	32 s/182 s	310 °C	305@100 ppm	[39]
HEA/Nb_2_O_5_	3 s/31 s	175 °C	28.5@400 ppm	[40]
ZnO film	60 s/90 s	RT	96@500 ppm	[41]
ZnO/Co_3_O_4_	11 s/32 s	RT	65.14@1000 ppm	This work

## Data Availability

The data in this article will be made available upon reasonable request to the corresponding author.
